# Prognostic Value of Atrial Phasic Dysfunction by CMR Feature Tracking for New-Onset Atrial Fibrillation in Patients with Cardiac Sarcoidosis

**DOI:** 10.3390/biomedicines14010185

**Published:** 2026-01-15

**Authors:** Nicoleta Nita, Johannes Mörike, Dominik Felbel, Rima Melnic, Felix von Sanden, Sascha d’Almeida, Wolfgang Rottbauer, Dominik Buckert

**Affiliations:** Department of Cardiology, Ulm University Heart Center, Albert-Einstein-Allee 23, 89081 Ulm, Germany

**Keywords:** atrial dysfunction in cardiac sarcoidosis, atrial fibrillation in cardiac sarcoidosis, left atrial reservoir strain in cardiac sarcoidosis

## Abstract

**Background/Objectives**: It is unclear whether assessment of phasic atrial function could improve risk stratification for new-onset atrial fibrillation (AF) in patients with newly diagnosed cardiac sarcoidosis (CS). We aimed to investigate the prognostic value of left atrial (LA) phasic dysfunction by cardiac magnetic resonance (CMR) for new-onset AF in newly diagnosed patients with CS. **Methods**: 78 patients with CS, without a prior history of AF, were studied using CMR feature tracking. Over a 4-year follow-up period, AF was documented by Holter monitoring and interrogation of intracardiac devices. Clinically silent CS was defined as CS in patients with biopsy-proven extracardiac sarcoidosis, with no cardiac symptoms, but with abnormalities on CMR or positron emission tomography consistent with CS. **Results**: Patients with clinically manifest CS were younger (mean age 56 vs. 51 years, *p* = 0.018), had poorer ventricular function, higher extent of atrial late gadolinium enhancement and significantly lower LA reservoir, conduit and booster function compared to patients with clinically silent CS. Over a 4-year follow-up period, 39% of patients with clinically manifest CS and 29.7% of patients with clinically silent CS developed AF. LA reservoir strain was a strong predictor of AF in the entire cohort. In subgroup analysis, age (HR 1.30, 95% CI 1.02–1.65, *p* = 0.030) and LA reservoir strain (HR 0.63, 95% CI 0.44–0.90, *p* = 0.011) were independent predictors of AF in patients with clinically silent CS, whereas baseline NT-proBNP (HR 1.003, 95% CI 1.001–1.006, *p* = 0.017) predicted AF in patients with clinically manifest CS. **Conclusions**: Reduced LA reservoir strain on CMR predicts new-onset AF in patients with newly diagnosed CS. The predictive value of LA reservoir strain is strongest in clinically silent CS and decreases with disease progression in clinically manifest CS.

## 1. Introduction

The prevalence of cardiac sarcoidosis (CS) has increased over the past decades due to growing awareness of the disease in the multimodality imaging era [1]. Despite the significant burden of atrial arrhythmias in CS [2,3,4,5], current guidelines contain recommendations limited to ventricular arrhythmias [6], and existing research on atrial fibrillation (AF) has mainly focused on patients with clinically manifest CS [5]. The extent of left atrial (LA) remodeling and the incidence of AF in clinically silent CS remain largely unknown. While the prognostic performance of phasic atrial function has been demonstrated in various cardiac conditions, including ischemic and non-ischemic cardiomyopathies [7,8], its predictive value for AF in patients with different clinical CS phenotypes is unclear. In the present study, we evaluated the incidence of AF and conducted a comprehensive assessment of phasic left atrial function by cardiovascular magnetic resonance (CMR) in patients with newly diagnosed CS. We also sought to determine whether indices of phasic atrial function could predict AF in patients with either clinically silent or clinically manifest CS.

## 2. Materials and Methods

### 2.1. Study Population

The study population included 78 consecutive patients with CS who were identified from a cohort of 621 patients with extracardiac sarcoidosis referred to the Heart and Lung Department of the University Hospital of Ulm between 2000 and 2021. All patients had either clinically manifest CS or clinically silent CS and underwent CMR within 6 months of CS diagnosis. Patients with a history of atrial fibrillation prior to the CMR examination were excluded from the current analysis. CS was diagnosed according to the Heart Rhythm Society criteria, based on either positive myocardial biopsy in patients with definite CS or on extracardiac histology with clinical and imaging involvement criteria in patients with probable CS [6]. Clinically silent CS was defined as CS in patients with biopsy-proven extracardiac sarcoidosis with abnormalities on screening ECG and echocardiography and typical findings for CS on imaging by CMR or positron emission tomography (PET), but no cardiac symptoms at the time of diagnosis, such as heart failure symptoms (New York Heart Association class ≥ II, peripheral edema), palpitations, arrhythmia, symptomatic atrioventricular block (dizziness, syncope), or chest pain. Typical CMR abnormalities indicative of CS included the presence of left ventricular late gadolinium enhancement (LGE) with a non-ischemic pattern and typical CS locations such as septal, intramural multifocal, or septal subepicardial, with or without LGE involvement of the right ventricular (RV) septum or RV free wall.

Demographic data, baseline clinical and therapeutic information, medical history and follow-up data, including clinical status, deaths and arrhythmic events, were retrospectively collected from the electronic medical records of the University Hospital of Ulm. Data on ventricular and atrial 18F-FDG uptake were retrospectively collected from available PET reports for 32 patients who underwent the investigation within 6 months of CS diagnosis. For a comprehensive assessment of phasic atrial function in sarcoidosis, patients with CS were compared with an age- and sex-matched cohort of healthy controls. The median follow-up time was 4.2 years from the CMR examination. The endpoint of interest was the occurrence of AF. Documentation of AF was obtained from 12-lead electrocardiograms (ECGs), short-term (24 h–72 h) and long-term (7 days) Holter monitors, event recorders and ICD or pacemaker interrogation. Patients without cardiac devices underwent ECG Holter monitoring at least once a year during the annual clinical follow-up. Documented arrhythmic events were adjudicated according to current guidelines [9].

This study was approved by the ethical board of the University of Ulm (approval number 236/18) and complied with the Declaration of Helsinki.

### 2.2. CMR Protocol

Patients underwent CMR examinations using a protocol designed for inflammatory cardiac disease on a clinical 1.5 Tesla scanner (Achieva, Philips Healthcare, Best, The Netherlands). Multi-slice b-SSFP Cartesian sequences and retrospective ECG gating were performed to cover the entire cardiac cycle with 32 phases and following acquisition parameters: temporal resolution of ~30 ms at a nominal heart rate of 60 bpm, repetition time 2.42 ms, echo time 1.2 ms, flip angle 60°, field of view 380 × 380 mm^2^, in plane resolution 1.4 × 1.4 mm^2^, slice thickness of 8 mm. If a higher temporal resolution was required, depending on the heart rate, more output phases were generated, albeit at the expense of a longer acquisition time. Ventricular LGE acquisitions were performed in all subjects using an extracellular contrast agent (Gadovist, Bayer Healthcare, Leverkusen, Germany) with segmented inversion-recovery gradient sequences performed in the long and short axes according to the recommendations of the Society of Cardiac Magnetic Resonance [10]. LA LGE acquisitions were performed in 21 patients according to the recommendations of previous studies [11].

#### Volumetry and Feature Tracking Analysis

All measurements, including ventricular and atrial volumetry, LGE assessment and atrial CMR-FT were performed using the software CVI^42^ v6.0(Circle Cardiovascular Imaging Inc., Calgary, AB, Canada). Left atrial phasic volumes were obtained from the 4-chamber and 2-chamber long-axis views. Maximal volume (V_max_) was obtained at the end of the systolic phase of the cardiac cycle, just before the mitral valve opened. The pre-atrial volume (V_preA_) was measured just before atrial contraction and the minimal volume (V_min_) was measured at late end-diastole, when the atrial volume was at its lowest following atrial contraction before mitral valve closure. Atrial phasic volumes and functions were calculated using the following formulae [12]: total ejection fraction as equivalent for reservoir function = (V_max_ − V_min_)/V_max_ × 100%; passive ejection fraction expressing conduit function = (V_max_ − V_preA_)/V_max_ × 100%; active ejection fraction for booster function = (V_preA_ − V_min_)/V_preA_ × 100%. All volumes were adjusted for body surface area. Left atrial reservoir, conduit and pump strains and strain rates (SR) were calculated using two- and four-chamber views in accordance with previous studies recommendations [12,13,14].

### 2.3. Reproducibility Analysis 

Interobserver reproducibility of strain measurements was tested in 20 randomly selected individuals and was found to be good (Kappa coefficient 0.76).

### 2.4. Statistics

Statistical analyses were performed using IBM SPSS Statistics, version 24.0 (IBM Corporation, Armonk, New York, NY, USA). Continuous variables with a normal distribution were expressed as mean ± standard deviation (SD), or as median with the interquartile range (IQR) in cases of non-normal distribution. Categorical variables were presented as absolute numbers and percentages. Comparisons between subgroups were performed using a *t*-test or a Mann–Whitney U test for continuous variables or using a chi square test for categorical variables. Receiver-operating characteristic (ROC) analysis was used to determine the area under the curve (AUC) and establish whether various atrial parameters could predict AF. Optimal cut-off values for relevant parameters having an AUC above 0.7 were generated from the ROC analysis using the Youden threshold. The cumulative incidence of AF was estimated using Kaplan–Meier analysis, and log-rank tests were performed to assess differences between subgroups. Multivariable Cox regression analysis was performed to assess the influence of relevant parameters on AF. The algorithm was applied to all potentially relevant variables, including parameters from univariate logistic regression analysis with *p* < 0.10. Each regression model performed on the entire cohort included a maximum of four potential predictors, given the reduced number of AF events [15]. Regression models performed for subgroups included a maximum of three variables for patients with clinically manifest CS and two variables for those with clinically silent CS, ensuring a minimum of five outcome events per predictor variable [15]. Collinearity between parameters was analyzed using variance inflation factors. A two-tailed *p* < 0.05 was considered statistically significant.

## 3. Results

### 3.1. Patient Characteristics

In the entire cohort, 13 patients (16.7%) had definite CS, whereas 83.3% of patients had probable CS. 3 patients had isolated CS without extracardiac involvement. Table 1 and Table S1 summarize baseline demographic, clinical and laboratory data. The mean age was 53 ± 10 years, and 58.5% were women in the entire cohort. Patients with clinically manifest CS were significantly younger than those with clinically silent CS (mean age 56 vs. 51 years, *p* = 0.018) and presented significantly higher NT-proBNP levels at baseline (588 ± 343 ng/L vs. 317 ± 143 ng/L, *p* < 0.001). The burden of comorbidities, especially the prevalence of risk factors for AF, did not differ significantly between the two groups.

### 3.2. Results of Multimodality Cardiac Imaging

Table 2 summarizes the results of cardiac imaging, including CMR and PET-CT scans. Patients with clinically manifest CS had significantly lower left and right ventricular ejection fractions (LVEF: 47 ± 7% vs. 58 ± 4%, *p* < 0.001) and significantly larger ventricles compared with patients with clinically silent CS. Immunosuppressive therapy, either corticosteroids or other immunosuppressants including azathioprine and methotrexate, was used at the time of CMR in 9 patients (22%) with clinically manifest CS and 5 patients (13.5%) with clinically silent CS. At the time of CS diagnosis, none of the patients had specific antiarrhythmic medication. Patients with clinically manifest CS had a higher prevalence of LA LGE on CMR and atrial 18F-FDG uptake on PET. Patients with clinically manifest CS had a significantly higher extent of LV LGE than patients with clinically silent CS (17% ± 4 vs. 12% ± 3, *p* < 0.001), whereas the prevalence of ventricular 18F-FDG uptake was similar between the 2 groups (78.6% vs. 72.2%, *p* = 0.681). None of the patients had isolated LA LGE or atrial 18F-FDG uptake without ventricular involvement.

### 3.3. Patterns of Phasic Left Atrial Function in Patients with Clinically Silent and Clinically Manifest CS

Compared with healthy controls, patients with clinically silent CS had significantly lower LA reservoir and conduit function but preserved LA booster function. The LA maximum volume index was not significantly different between patients with clinically silent CS and healthy controls. However, the LA pre-contractile volume index and LA minimum volume index were significantly higher in patients with clinically silent CS compared to healthy controls (Table S2).

Table 3 shows a detailed comparison of left atrial reservoir, conduit and booster function between patients with clinically silent and clinically manifest CS. Patients with clinically manifest CS had significantly lower strain parameters of reservoir, conduit, and booster function compared to patients with silent CS. Overall, 39.7% of patients presented reduced LA reservoir strain at baseline, below the cut-off of 21.2%. Within the subgroups, 29.7% of patients with clinically silent CS and 48.8% of patients with clinically manifest CS had reduced LA reservoir strain < 21.2%.

### 3.4. Incidence of Atrial Arrhythmia and Clinical Management

All patients with clinically manifest CS and 62.2% of patients with clinically silent CS received corticosteroids after diagnosis of CS. A total of 6 patients died during follow-up (4 patients with clinically manifest CS and 2 patients with clinically silent CS), 1 patient received a heart transplantation and sustained ventricular tachycardia occurred in 18 patients (16.2% of patients with clinically silent CS and 29.3% of patients with clinically manifest CS). A total of 45 patients received a pacemaker and/or implantable defibrillator during follow-up (68.3% of patients with clinically manifest CS and 45.9% of patients with clinically silent CS).

Over a median follow-up time of 4.2 years from the CMR examination, AF was documented in 27 patients (34.6% of the study population). Within the subgroups, 29.7% of patients with clinically silent CS and 39% of patients with clinically manifest CS developed AF at 4-year follow-up. AF was diagnosed by 12-lead ECG or by Holter in 34.6% of cases and by cardiac device interrogation in 65.4% of cases. Recurrent AF episodes were documented in 9 patients (33.4%), while 66.6% of patients had only one documented AF episode. Within the subgroup with clinically silent CS, the incidence of AF at 4 years was significantly lower in patients treated with corticosteroids after CS diagnosis than in patients not treated with corticosteroids after CS diagnosis (17.4% vs. 50%, *p* = 0.035). 52.6% of patients in the entire cohort received oral anticoagulation. Most patients with AF were asymptomatic (73%), whereas rhythm control was required by electrical cardioversion in 6 patients (7.7%), catheter ablation in 6 patients (7.7%) and antiarrhythmic drugs, including amiodarone, in 9 patients (11.5%). No embolic ischemic events were documented during follow-up.

### 3.5. Predictors of Atrial Fibrillation

In the ROC analysis, LA reservoir strain followed by LA total function showed the best predictive value for AF at follow-up (Table S3, Figure S1). An optimized cut-off value of 21.2% for LA reservoir strain was identified as predictive of AF.

Table S4 and Table 4 show the results of the univariate and multivariable Cox regression analyses in the entire cohort. Age, NT-proBNP, and LA reservoir strain were statistically significantly predictive of AF by multivariable Cox regression analysis (Table 4). In the subgroup analysis with univariate and multivariate Cox regression analysis, LA reservoir strain and LA total function were consistent independent predictors for AF in patients with clinically silent CS, whereas in patients with clinically manifest CS, the LA phasic function parameters were not predictive of AF (Table 5 and Table 6, Tables S5 and S6). Age was significantly predictive of AF in both subgroups, whereas elevated NT-proBNP was an independent predictor of AF only in patients with clinically manifest AF.

**Figure 1:**
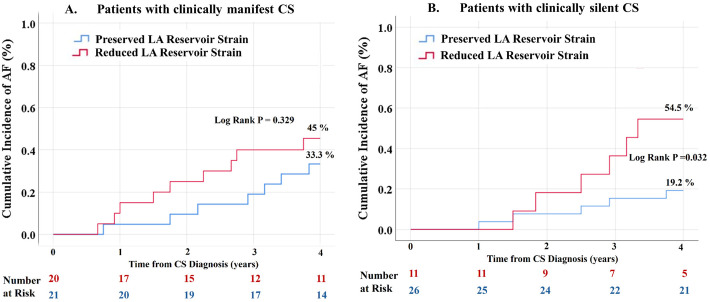
Incidence of atrial fibrillation in relation to LA reservoir strain in patients with clinically manifest and clinically silent cardiac sarcoidosis.

Figure 1 shows the cumulative incidence of AF at 4 years stratified by the presence of reduced LA reservoir strain (cut-off value 21.2%) for patients with clinically manifest CS (Figure 1A) and clinically silent CS (Figure 1B). Within the subgroup with clinically silent CS, patients with reduced LA reservoir strain at baseline had a significantly higher incidence of AF at 4 years compared to patients with preserved LA reservoir strain (54.5% vs. 19.2%, log-rank *p* = 0.032). In patients with clinically manifest CS, there was a trend towards a higher incidence of AF at 4 years in patients with reduced LA reservoir strain at baseline, but the difference was not statistically significant (45% vs. 33.3%, log-rank *p* = 0.329). In patients with clinically silent CS, the use of corticosteroids after CS diagnosis was significantly associated with the occurrence of AF in the univariate analysis HR 0.21 (95% CI 0.05–0.95), *p* = 0.042. However, due to the small number of patients and reduced number of AF events in this subgroup, the use of corticosteroids was not included in the multivariable analysis.

Incidence curves derived from Kaplan–Meier analysis in patients with clinically manifest CS (A.) and clinically silent CS (B.), stratified by the presence of reduced LA reservoir strain at baseline. Reduced LA reservoir strain was defined as LA reservoir strain < 21.2%. In clinically silent CS, the cumulative incidence of AF was statistically higher in patients with reduced LA reservoir strain at baseline compared to patients with preserved LA reservoir strain. In clinically manifest CS, there was a trend towards a higher AF incidence in patients with reduced LA reservoir strain at baseline, but this did not reach statistical significance; AF = atrial fibrillation; CS = cardiac sarcoidosis; LA = left atrial.

## 4. Discussion

In the present study, we performed a comprehensive assessment of phasic left atrial function by CMR feature tracking and evaluated cumulative incidences of AF in different clinical phenotypes of CS. Reduced LA reservoir strain is an indicator of adverse atrial remodeling and was a strong independent predictor of AF in patients with CS. The predictive value of LA reservoir strain is highest in patients with clinically silent CS but decreases with disease progression in patients with clinically manifest CS who present with advanced atrial cardiomyopathy.

### 4.1. Incidence of AF in Different CS Phenotypes

Our study showed a cumulative risk of AF at 4 years from diagnosis of 39% in patients with clinically manifest CS and 29.7% in patients with clinically silent CS. Several studies previously reported overall prevalences of atrial arrhythmias (AF and non-AF) in mixed cohorts with CS, and only one study reported time-to-AF data for cumulative incidence in patients with clinically manifest CS [5]. Time-to-AF cumulative incidences in patients with clinically silent CS have not been reported, given that most studies focused on clinically manifest CS, and clinically silent CS has remained in the background of clinical research. The prevalence of AF in clinically manifest CS ranged from 16% to 37% in previous studies [2,16,17]. Niemela et al. reported an AF incidence of 29% 3 years after CS diagnosis in a larger cohort of 118 patients with clinically manifest CS, which is lower than in our cohort with clinically manifest CS. Patients with clinically manifest CS in the present study appear to be at a more advanced stage of disease, with a higher prevalence of heart failure symptoms at the time of diagnosis of 14.6%. A notable finding of the present study is the high incidence of AF in patients with clinically silent CS, which is comparable to the incidence of AF in clinically manifest CS reported in other studies [5]. Although most patients with clinically silent CS had asymptomatic AF episodes during follow-up, 21.6% of the patients required rhythm control, highlighting the importance of screening for CS and AF in patients with extracardiac sarcoidosis.

### 4.2. Multimodality Imaging

The presence of atrial involvement on CMR LA LGE sequences was significant and comparable in patients with clinically silent and clinically manifest CS (33.3% and 41.7%, respectively). 38.9% of patients with clinically manifest CS had atrial ^18^F-FDG uptake, which is comparable to prevalence rates reported in previous studies [2,5]. Although the prevalence of atrial ^18^F-FDG uptake was higher in patients with clinically manifest CS, it was not statistically significantly different from that in patients with clinically silent CS. These findings suggest extensive atrial inflammation in patients with clinically silent CS, which might explain the high incidence of future AF.

### 4.3. Patterns of Phasic LA-Atrial Function

Substantial data previously demonstrated the prognostic significance of phasic LA emptying fractions and strains in various cardiac diseases, yet it is unclear whether patients with different phenotypes of CS would benefit from systematic LA strain measurements by CMR feature tracking at the time of CS diagnosis. Reduced LA strain was a prevalent finding in both patients with clinically manifest CS (48.8%) and patients with clinically silent CS (29.7%). Within the subgroup with clinically silent CS, patients with reduced reservoir strain had a significantly lower incidence of AF during follow-up than patients with preserved LA reservoir strain. LA reservoir function is related to a fine interplay between atrial compliance and LV systolic motion of the basal ventricular walls, which are a predilect area of infiltration in cardiac sarcoidosis. Thus, reduced LA reservoir strain in CS is a consequence of either atrial wall inflammation with consequent scarring or ventricular dysfunction. Notably, booster pump function was preserved in patients with clinically silent CS. Preserved or even increased atrial pump function has been observed in patients with early stages of hypertension, diabetes mellitus and in younger obese adults as a compensatory mechanism for the impaired conduit and reservoir function [18,19,20]. However, with progressive elevation of ventricular filling pressures, booster atrial function decreases and is associated with the onset of heart failure symptoms [21,22].

### 4.4. Clinical Implications

Measurement of LA phasic function at the time of CS diagnosis can detect subtle atrial dysfunction and may improve risk stratification for future AF, especially in patients with clinically silent CS, who usually present at an earlier stage of the disease with normal LV ejection fraction and preserved LA booster function. Patients with clinically manifest CS present with advanced atrial and ventricular scarring. In these patients, NT-proBNP at baseline was a consistent predictor of AF in our study, and elevated levels at the time of diagnosis should prompt screening for AF. Current guidelines lack consensus on the management of clinically silent CS [16]. Our observation of a lower incidence of AF in patients with clinically silent CS treated with corticosteroids after CS diagnosis suggests that corticosteroids may prevent extensive atrial inflammation and scarring, which are known to trigger AF. These findings need to be confirmed in future larger studies in patients with clinically silent CS.

### 4.5. Strengths and Limitations

Compared to previous research, our study is the only one with analysis of time-to-AF data in patients with clinically silent CS and provides a comprehensive characterization of atrial remodeling in different phenotypes of CS, using multimodality imaging with LA LGE, phasic atrial strain and PET-CT. A limitation of this study is the small sample size, especially in the subgroup with clinically silent CS. This is a common problem in CS studies, which underlines the need for future multicentric studies to ensure a larger sample size to confirm our findings. The present analysis is based on a population from a single tertiary care center, and the results may not be fully representative of the broader cardiac sarcoidosis population. LA LGE and PET examinations at baseline were available in a reduced number of patients, as LA LGE sequences have only been introduced into the CS CMR protocol in recent years, and PET examinations were rather performed during follow-up to assess disease activity and response to immunosuppressive therapy. Therefore, both baseline LA LGE and atrial ^18^F-FDG uptake as potentially important risk factors for AF, especially in patients with manifest CS, were not included in the multivariable regression analysis. The low AF recurrence rate in this study may be underestimated due to inconsistent follow-up and lack of serial ECG Holter testing in patients without intracardiac devices. Finally, this was a retrospective study that included patients over a long period of time of 22 years, and some important data, such as NT-proBNP, were missing in patients enrolled in the early years.

## 5. Conclusions

Over a follow-up period of 4 years, we report a significant cumulative incidence of AF in 39% of patients with clinically manifest CS and 29.5% of patients with clinically silent CS. Given the high incidence of atrial fibrillation in patients with both silent and manifest cardiac sarcoidosis, early identification of patients at risk of developing atrial fibrillation may improve management and long-term outcomes. Reduced LA reservoir strain is a prevalent finding in patients with CS with a strong predictive value for new-onset AF, especially in patients with clinically silent CS who present at earlier stages of the disease. The predictive value of atrial strain decreases in patients with clinically manifest CS who show advanced atrial cardiomyopathy at baseline. Therefore, risk stratification in CS should be individualized according to the clinical phenotype at diagnosis, as some patients may benefit more from phasic atrial assessment. The potential of corticosteroids to reduce the risk of AF in clinically silent CS should be evaluated in larger studies.

## Figures and Tables

**Table 1:** Baseline Characteristics.

	Clinically Silent CS (*N* = 37)	Clinically Manifest CS (*N* = 41)	*p* Value
Age	56 ± 10	51 ± 8	0.018
Female	21 (56.8)	26 (63.4)	0.549
Body Mass Index	1.9 ± 0.2	2 ± 0.22	0.175
**Comorbidities**			
Hypertension	11 (29.7)	11 (26.8)	0.776
Diabetes Mellitus	3 (8.1)	6 (14.6)	0.368
Coronary artery disease	2 (5.4)	3 (7.3)	0.731
Sleep apnea	3 (8.1)	4 (9.8)	0.799
Hyperthyroidism	0	1 (2.4)	0.339
Extracardiac sarcoidosis	37 (100)	38 (92.7)	0.093
**Clinical cardiac manifestations**			
Atrio-ventricular Block	0	22 (53.7)	<0.001
Ventricular Tachyarrhythmia	0	8 (19.5)	0.005
Heart Failure	0	6 (14.6)	0.005
Other	0	7 (17.1)	0.008
**Laboratory values**			
Troponin T, pg/mL	8 ± 3.2	15 ± 9.5	<0.001
NT-proBNP, ng/L	317 ± 143	588 ± 343	<0.001
GFR, mL/min	77 ± 18	74 ± 15	0.463

Values are *n* (%) or mean ± SD. CS = cardiac sarcoidosis; GFR = glomerular filtration rate; NT-proBNP = N-terminal pro B-type natriuretic peptide.

**Table 2:** Multimodality imaging.

	Clinically Silent CS (*N* = 37)	Clinically Manifest CS (*N* = 41)	*p* Value
**CMR**			
LA LGE ^a^	3/9 (33.3)	5/12 (41.7)	0.697
LVEDVI, mL/m^2^	88 ± 14	99 ± 13	<0.001
LVEF, %	58 ± 4	47 ± 7	<0.001
LV Mass, g/m^2^	62 ± 14	64 ± 16	0.503
LGE extent, % of LV mass	12 ± 3	17 ± 4	<0.001
LV GLS, %	12.9 ± 1.54	11.4 ± 1.62	<0.001
RVEDVI, mL/m^2^	85 ± 13	94 ± 16	0.009
RVEF, %	57 ± 4	53 ± 7	0.001
**PET ^b^**			
LA ^18^F-FDG uptake	3/14 (21.4)	7/18 (38.9)	0.290
LV ^18^F-FDG uptake	11/14 (78.6)	13/18 (72.2)	0.681

Values are *n* (%) or mean ± SD. CMR = cardiac magnetic resonance; ^18^F-FDG = ^18^F-Fluorodeoxyglucose; GLS = global longitudinal strain; LA = left atrial; LGE = late gadolinium enhancement; LV = left ventricle; LVEDVI = left ventricular end diastolic volume index; LVEF = left ventricular ejection fraction; PET = positron emission tomography; RVEDVI = right ventricular end diastolic volume index; RVEF = right ventricular ejection fraction; ^a^ atrial LGE sequences were available for 21 patients in total; ^b^ PET was available for 32 patients.

**Table 3:** Atrial phasic function by CMR feature tracking.

	Clinically Silent CS (*N* = 37)	Clinically Manifest CS (*N* = 41)	*p* Value
LAV max, mL/m^2^	36 ± 7	40 ± 6	0.013
LAV min, mL/m^2^	18 ± 4	20 ± 5	0.010
LAV preA, mL/m^2^	27 ± 5	29 ± 6	0.052
**LA Reservoir function**			
LA total EF, %	52 ± 6	48 ± 6	0.001
LA reservoir strain, %	26.7 ± 8.1	21.4 ± 5.0	0.001
LA reservoir strain rate, s^−1^	1.4 ± 0.4	1.1 ± 0.4	0.001
**LA Conduit function**			
LA passive EF, %	37 ± 9	32 ± 6	0.003
LA conduit strain, %	13.3 ± 5.6	10.3 ± 4.4	0.009
LA conduit strain rate, s^−1^	1.6 ± 0.4	1.3 ± 0.4	0.001
**LA Booster function**			
LA active EF, %	35 ± 6	32 ± 6	0.055
LA booster strain, %	11.9 ± 1.4	11 ± 1.6	0.017
LA booster strain rate, s^−1^	1.6 ± 0.5	1.3 ± 0.5	0.017

Values are *n* (%) or mean± SD; LA = left atrial volume; LAV max = maximum left atrial volume; LAV min = minimum left atrial volume; LAV preA = pre-atrial contraction left atrial volume.

**Table 4:** Multivariable predictors of atrial fibrillation in the entire cohort.

	Model 1 (*n* = 78, e = 27)		Model 2 (*n* = 78, e = 27)		Model 3 (*n* = 78, e = 27)	
	HR (95% CI)	*p* Value	HR (95% CI)	*p* Value	HR (95% CI)	*p* Value
**Age**	1.23 (1.09–1.37)	<0.001	1.24 (1.10–1.39)	<0.001	1.21 (1.09–1.35)	0.001
**Hypertension**	4.78 (0.84–27.2)	0.078	-	N/A	4.24 (0.86–21.19)	0.078
**NT-pro-BNP**	1.004 (1.001–1.007)	0.009	1.004 (1.001–1.007)	0.011	-	N/A
**LA Reservoir Strain (%)**	0.77 (0.67–0.89)	0.001	0.76 (0.65–0.89)	0.001	0.79 (0.69–0.91)	0.001
**La Total Function (%)**	-	N/A	0.88 (0.78–1.00)	0.061	0.89 (0.79–1.00)	0.062

Multivariable Models based on Cause-Specific Cox Regression Analyses. e = number of events; LA = left atrium; *n* = number of patients; N/A = not applicable.

**Table 5:** Multivariable predictors of atrial fibrillation in clinically manifest cardiac sarcoidosis.

	Model 1 (*n* = 41, e = 16)		Model 2 (*n* = 41, e = 16)		Model 3 (*n* = 41, e = 16)	
	HR (95% CI)	*p* Value	HR (95% CI)	*p* Value	HR (95% CI)	*p* Value
**Age**	-	N/A	1.16 (1.04–1.32)	0.011	1.16 (1.04–1.30)	0.010
**Hypertension**	3.54 (0.60–20.8)	0.161	-	N/A	-	N/A
**NT-proBNP**	1.003 (1.001–1.006)	0.017	1.003 (1.001–1.006)	0.047	1.003 (1.001–1.006)	0.048
**LA Reservoir Strain (%)**	0.89 (0.76–1.05)	0.179	0.86 (0.72–1.03)	0.110	-	N/A
**LA Total Function (%)**	-	N/A	-	N/A	0.92 (0.80–1.06)	0.260

Multivariable Models based on Cause-Specific Cox Regression Analyses. e = number of events; LA = left atrium; *n* = number of patients; N/A = not applicable.

**Table 6:** Multivariable predictors of atrial fibrillation in clinically silent cardiac sarcoidosis.

	Model 1 (*n* = 37, e = 11)		Model 2 (*n* = 37, e = 11)		Model 3 (*n* = 37, e = 11)	
	HR (95% CI)	*p* Value	HR (95% CI)	*p* Value	HR (95% CI)	*p* Value
**Age**	1.30 (1.02–1.65)	0.030	-	N/A	-	N/A
**Hypertension**	-	N/A	6.71 (0.53–84.23)	0.140	-	N/A
**LA Reservoir Strain (%)**	0.63 (0.44–0.90)	0.011	-	N/A	0.72 (0.54–0.97)	0.030
**LA Total Function (%)**	-	N/A	0.68 (0.51–0.89)	0.007	0.73 (0.54–0.99)	0.042

Multivariable Models based on Cause-Specific Cox Regression Analyses. e = number of events; LA = left atrium; *n* = number of patients; N/A = not applicable.

## Data Availability

The data underlying this article will be shared upon reasonable request to the corresponding author. The data are not publicly available due to data privacy laws.

## Supplementary Materials

The following supporting information can be downloaded at: https://www.mdpi.com/article/10.3390/biomedicines14010185/s1, Figure S1: Risk prediction for atrial fibrillation using ROC analysis; Table S1: Baseline characteristics stratified by occurrence of AF at follow-up; Table S2: Atrial phasic function by CMR in patients with clinically silent CS vs. healthy controls; Table S3: Risk prediction for atrial fibrillation with ROC analysis; Table S4: Univariate Analysis for prediction of incident AF in the entire cohort; Table S5: Univariate Analysis for prediction of incident AF in clinically manifest CS; Table S6: Univariate Analysis for prediction of incident AF in clinically silent CS.

## Abbreviations

The following abbreviations are used in this manuscript:

AF	atrial fibrillation
CMR-FT	cardiac magnetic resonance imaging feature tracking
CS	cardiac sarcoidosis
ECG	Electrocardiogram
LA	left atrium
LV-LGE	left ventricular late gadolinium enhancement
