# Supplementary material for: Prognostic Value of Atrial Phasic Dysfunction by CMR Feature Tracking for New-Onset Atrial Fibrillation in Patients with Cardiac Sarcoidosis
Source: Biomedicines. 2026 Jan 15;14(1):185. doi: 10.3390/biomedicines14010185 (PMC12838861; doi:10.3390/biomedicines14010185)
Supplement: Supplementary file 1 [file biomedicines-14-00185-s001.zip › biomedicines-4084258-supplementary.pdf]

Table S1. Baseline characteristics stratified by occurrence of AF at follow-up

	No Atrial fibrillation (N=51)	Atrial Fibrillation (N=27)	*P* Value
Age	50±8	60±10	<0.001
Female	29(56.9)	18 (66.7)	0.400
Comorbidities			
Hypertension	8(15.7)	13(48.1)	0.002
Diabetes Mellitus	4(7.8)	5(18.5)	0.160
Coronary artery disease	4(7.8)	1(3.7)	0.478
Sleep apnea	3(5.9)	4(14.8)	0.189
Hyperthyroidism	1(2)	0(0)	0.464
Laboratory values			
Troponin T, pg/ml	11±8	13±8	0.228
NT-pro BNP, ng/l	385±243	615±349	0.001
GFR, ml/min	76±15	77±19	0.759
Imaging Variables			
LA LGE[a]	3/14 (21.4)	5/7 (71.4)	0.026
LVEDVI, ml/m^2	95±14	90±14	0.124
LVEF, %	55±7	52±8	0.139
LV global longitudinal strain, %	12.3±1.7	11.9±1.8	0.382
LGE extent, % of LV mass	14±4	15±5	0.550
RVEDVI, ml/m^2	90±15	89±17	0.746
RVEF, %	55±5	53±6	0.121
LV ^{18}F-FDG uptake[b]	13/18 (72.2)	11/14 (78.6)	0.681
LA ^{18}F-FDG Uptake[b]	2/18(11.1)	8/14(57.1)	0.005
LAV max, ml/m^2	37±7	38±5	0.651
LAV min, ml/m^2	18±4	20±5	0.102
LAV preA, ml/m^2	28±5	29±5	0.170
LA total EF, %	52±5	47±7	0.001
LA passive EF, %	36±8	31±6	0.004
LA active EF, %	34±5	31±8	0.018
LA reservoir strain, %	26.2±7.2	19.6±4.8	<0.001
LA conduit strain, %	12.9±5.6	9.5±3.5	0.005
LA booster strain, %	11.7±1.4	10.8±1.8	0.018

Values are n (%) or mean± SD. AF= atrial fibrillation; CS = cardiac sarcoidosis; GFR = glomerular filtration rate; 18F-FDG =18F-Fluorodeoxyglucose; LA=left atrial; LAV max = maximum left atrial volume; LAV min= minimum left atrial volume; LAV preA = pre-atrial contraction left atrial volume; LGE = late gadolinium enhancement; LV=left ventricle; LVEDVI = left ventricular end diastolic volume index; LVEF = left ventricular ejection fraction; NT-proBNP = N-terminal pro B-type natriuretic peptide; RVEDVI = right ventricular end diastolic volume index; RVEF = right ventricular ejection fraction; [a] atrial LGE sequences were available for 21 patients in total; [b] PET was available for 32 patients.

Table S2. Atrial phasic function by CMR in patients with clinically silent CS vs healthy controls

	**Clinically Silent CS (N=37)**	**Healthy Controls (N=37)**	***P* Value**
LAV max (ml/m^2)	36±7	35±5	0.605
LAV min (ml/m^2)	18±4	13±3	<0.001
LAV preA (ml/m^2)	27±5	21±4	<0.001
LA Reservoir function			
LA total EF (%)	52±6	63±5	<0.001
LA reservoir strain (%)	26.7±8.1	33.9±9.4	0.001
LA reservoir strain rate (s^{-1})	1.4±0.4	2.1±0.4	<0.001
LA Conduit function			
LA passive EF (%)	37±9	41±5	0.027
LA conduit strain (%)	13.3±5.6	21.4±5.9	<0.001
LA conduit strain rate (s^{-1})	1.6±0.4	2.2±0.4	<0.001
LA Booster function			
LA active EF (%)	35±6	37±6	0.082
LA booster strain (%)	11.9±1.4	12.5±4.3	0.402
LA booster strain rate (s^{-1})	1.6±0.5	1.6±0.5	0.927

Values are n (%) or mean± SD; LA= left atrial volume; LAV max = maximum left atrial volume; LAV min= minimum left atrial volume; LAV preA= pre-atrial contraction left atrial volume.

Table S3. Risk prediction for atrial fibrillation with ROC analysis

	AUC	CI	***P* Value**
LA Reservoir Strain	0.756	0.650-0.863	<0.001
LA Total Function	0.752	0.627-0.878	<0.001
LA Passive Function	0.696	0.573-0.818	0.005
LA Conduit Strain	0.687	0.567-0.807	0.007
LA Active Function	0.646	0.495-0.796	0.035
LA Booster Strain	0.662	0.520-0.804	0.019
LAV min, ml/m^2	0.613	0.474-0.752	0.102
LAV preA, ml/m^2	0.588	0.454-0.722	0.202
LAV max, ml/m^2	0.535	0.406-0.664	0.610

AUC = aria under the curve; CI = confidence interval; LA = left atrial; LAV max = maximum left atrial volume; LAV min = minimum left atrial volume; LAV preA = pre-atrial contraction left atrial volume; ROC = Receiver Operating Characteristic.

Table S4. Univariate Analysis for prediction of incident AF in the entire cohort

	**HR (95% CI)**	***P* Value**
Demographics/Comorbidities		
Age	1.13(1.06-1.19)	<0.001
Gender	1.16(0.57-4.01)	0.401
Hypertension	4.99 (1.72-14.51)	0.003
Sleep apnea	2.78(0.58-13.47)	0.203
Laboratory Variables		
NT-pro-BNP, pg/ml	1.003 (1.001-1.005)	0.003
Troponin, ng/l	1.04(0.98-1.10)	0.232
GFR, ml/min	1.005(0.98-1.03)	0.756
Imaging Variables		
LA Reservoir Strain, %	0.84 (0.76-0.92)	<0.001
La total function, %	0.86(0.78-0.94)	0.002
LAV max, ml/m^2	1.02(0.95-1.09)	0.647
LAV min, ml/m^2	1.09(0.98-1.21)	0.104
LAV preA, ml/m^2	1.07(0.97-1.17)	0.170
LA LGE	9.16(1.14-73.23)	0.037
LA ^{18}F-FDG Uptake	11.76(2.56-54.07)	0.002
LVEF, %	0.95(0.89-1.01)	0.142
LV LGE extent, of LV mass	1.03(0.93-1.14)	0.545
LV GLS, %	0.88 (0.67-1.17)	0.378
LV Mass, g/ m^2	1.02(0.99-1.05)	0.186
RVEF, %	0.93(0.85-1.02)	0.124
RVEDVI, ml/m^2	0.99(0.96-1.03)	0.742

AF = atrial fibrillation; 18F-FDG =18F-Fluorodeoxyglucose; GFR = glomerular filtration rate; HR= hazard ratio; LA= left atrium; LAV max = maximum left atrial volume; LAV min = minimum left atrial volume; LAV preA = pre-atrial contraction left atrial volume; LGE = late gadolinium enhancement; LV = left ventricle; LVEF = left ventricular ejection fraction; LV GLS = left ventricular global longitudinal strain; NT-proBNP = N-terminal pro B-type natriuretic peptide. RVEDVI = right ventricular end diastolic volume index; RVEF = right ventricular ejection fraction.

Table S5. Univariate Analysis for prediction of incident AF in clinically manifest CS

	**HR (95% CI)**	***P* Value**
Demographics/Comorbidities		
Age	1.18(1.06-1.32)	0.003
Gender	0.93(0.26-3.44)	0.923
Hypertension	5.70 (1.20-27.12)	0.029
Sleep apnea	1.64(0.20-13.01)	0.638
Laboratory Variables		
NT-pro-BNP, pg/ml	1.003 (1.001-1.005)	0.011
Troponin, ng/l	1.03(0.97-1.10)	0.336
GFR, ml/min	0.97(0.93-1.02)	0.248
Imaging Variables		
LA Reservoir Strain, %	0.92 (0.80-1.04)	0.189
La total function, %	0.92(0.82-1.04)	0.191
LAV max, ml/m^2	1.03(0.92-1.48)	0.630
LAV min, ml/m^2	1.07(0.93-1.23)	0.364
LAV preA, ml/m^2	1.08(0.96-1.20)	0.225
LA LGE	9.00(0.56-143.23)	0.120
LA ^{18}F-FDG Uptake	9.00(1.29-63.02)	0.027
LVEF, %	0.96(0.87-1.06)	0.428
LV LGE extent, of LV mass	1.12(0.94-1.33)	0.200
LV GLS, %	0.95(0.64-1.41)	0.794
LV Mass, g/ m^2	1.02(0.98-1.06)	0.383
RVEF, %	0.95(0.85-1.07)	0.404
RVEDVI, ml/m^2	0.99(0.95-1.03)	0.658

AF = atrial fibrillation; 18F-FDG =18F-Fluorodeoxyglucose; GFR = glomerular filtration rate; HR= hazard ratio; LA= left atrium; LAV max = maximum left atrial volume; LAV min = minimum left atrial volume; LAV preA = pre-atrial contraction left atrial volume; LGE = late gadolinium enhancement; LV=left ventricle; LV GLS = left ventricular global longitudinal strain; LVEF = left ventricular ejection fraction; NT-proBNP = N-terminal pro B-type natriuretic peptide. = right ventricular end diastolic volume index; RVEF = right ventricular ejection fraction.

Table S6. Univariate Analysis for prediction of incident AF in clinically silent CS

	**HR (95% CI)**	***P* Value**
Demographics/Comorbidities		
Age	1.16(1.04-1.29)	0.010
Gender	2.67(0.58-12.35)	0.210
Hypertension	5.04 (1.09-23.41)	0.039
Sleep apnea	5.55(0.45-68.42)	0.182
Laboratory Variables		
NT-pro-BNP, pg/ml	1.004 (0.999-1.009)	0.142
Troponin, ng/l	1.02(0.82-1.27)	0.856
GFR, ml/min	1.04(0.99-1.08)	0.109
Imaging Variables		
LA Reservoir Strain, %	0.70(0.54-0.92)	0.010
La total function, %	0.70 (0.54-0.90)	0.007
LAV max, ml/m^2	0.99(0.89-1.10)	0.883
LAV min, ml/m^2	1.12(0.93-1.35)	0.233
LAV preA, ml/m^2	1.04(0.88-1.21)	0.667
LA LGE	10.00(0.39-250.41)	0.161
LA ^{18}F-FDG Uptake	14.66(1.16-185.23)	0.038
LVEF, %	0.92(0.78-1.08)	0.311
LV LGE extent, of LV mass	0.82(0.64-1.06)	0.124
LV GLS, %	0.87(0.54-1.40)	0.558
LV Mass, g/ m^2	1.03(0.97-1.08)	0.349
RVEF, %	0.89(0.75-1.08)	0.252
RVEDVI, ml/m^2	0.98(0.93-1.05)	0.676
Use of corticosteroids after diagnosis	0.21(0.05-0.95)	0.042

AF = atrial fibrillation; CS = cardiac sarcoidosis; 18F-FDG = 18F-Fluorodeoxyglucose; GFR = glomerular filtration rate; HR= hazard ratio; LA= left atrium; LAV max = maximum left atrial volume; LAV min = minimum left atrial volume; LAV preA = pre-atrial contraction left atrial volume; LGE = late gadolinium enhancement; LV=left ventricle; LV GLS = left ventricular global longitudinal strain; LVEF = left ventricular ejection fraction; NT-proBNP = N-terminal pro B-type natriuretic peptide. LVEF = left ventricular ejection fraction; RVEDVI = right ventricular end diastolic volume index; RVEF = right ventricular ejection fraction.

Figure S1. Risk prediction for atrial fibrillation using ROC analysis

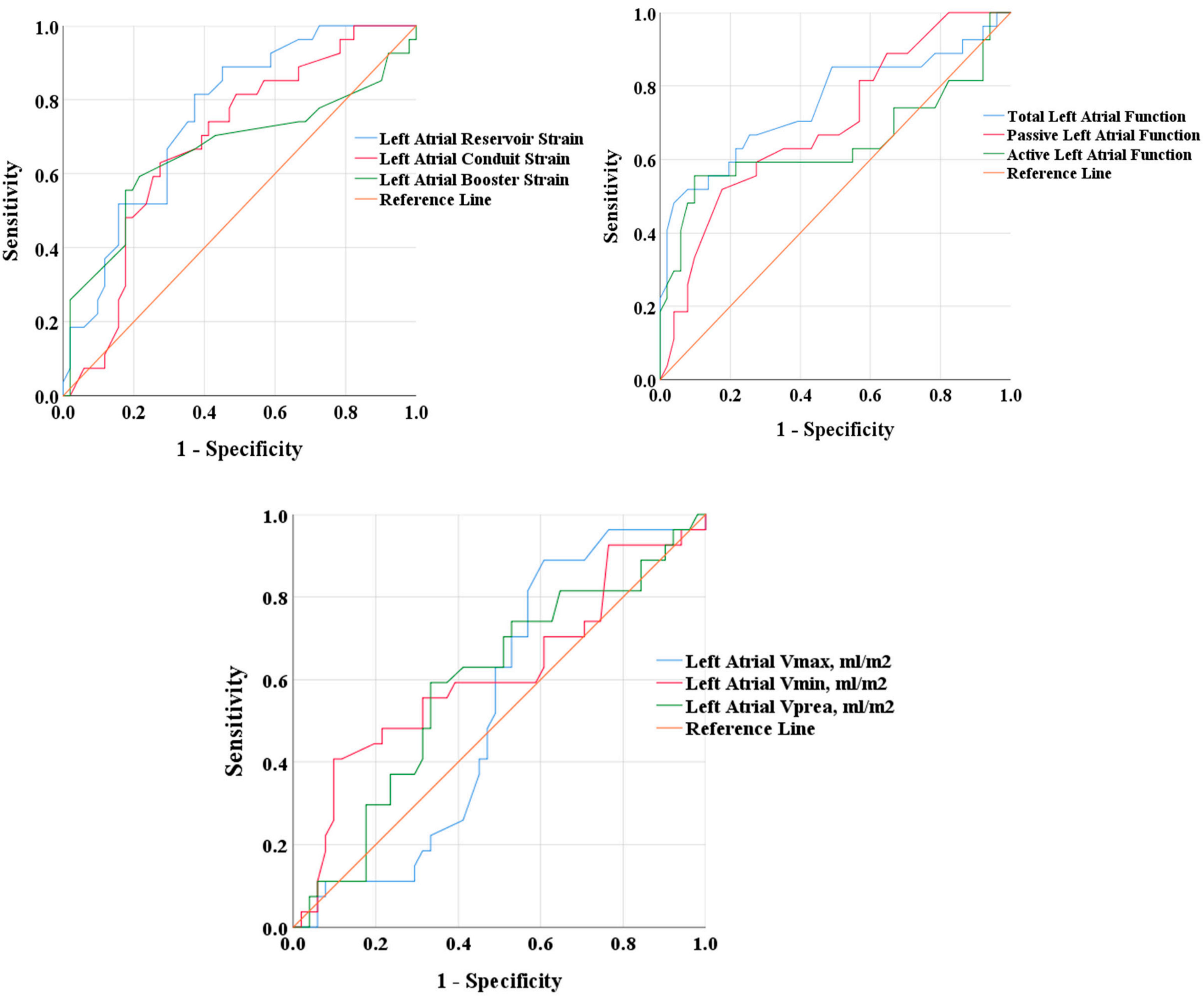

ROC = Receiver Operating Characteristic; Vmax= maximum volume; Vmin = minimum volume; VpreA = pre-atrial contraction volume.
